# hnRNPK promotes gastric tumorigenesis through regulating CD44E alternative splicing

**DOI:** 10.1186/s12935-019-1020-x

**Published:** 2019-12-12

**Authors:** Wei-zhao Peng, Ji-xi Liu, Chao-feng Li, Ren Ma, Jian-zheng Jie

**Affiliations:** 1 Department of General Surgery, China-Japan Friendship Hospital, Beijing, 100029 China; 2 Department of Gastroenterology, China-Japan Friendship Hospital, Beijing, 100029 China

**Keywords:** hnRNPK, Gastric cancer, SRSF1, CD44E, Alternative splicing

## Abstract

**Background:**

The high prevalence of alternative splicing among genes implies the importance of genomic complexity in regulating normal physiological processes and diseases such as gastric cancer (GC). The standard form of stem cell marker CD44 (CD44S) and its alternatives with additional exons are reported to play important roles in multiple types of tumors, but the regulation mechanism of CD44 alternative splicing is not fully understood.

**Methods:**

Here the expression of hnRNPK was analyzed among the Cancer Genome Atlas (TCGA) cohort of GC. The function of hnRNPK in GC cells was analyzed and its downstream targeted gene was identified by chromatin immunoprecipitation and dual luciferase report assay. Finally, effect of hnRNPK and its downstream splicing regulator on CD44 alternative splicing was investigated.

**Results:**

The expression of hnRNPK was significantly increased in GC and its upregulation was associated with tumor stage and metastasis. Loss-of-function studies found that hnRNPK could promote GC cell proliferation, migration, and invasion. The upregulation of hnRNPK activates the expression of the splicing regulator SRSF1 by binding to the first motif upstream the start codon (− 65 to − 77 site), thereby increasing splicing activity and expression of an oncogenic CD44 isoform, CD44E (has additional variant exons 8 to 10, CD44v8-v10).

**Conclusion:**

These findings revealed the importance of the hnRNPK-SRSF1-CD44E axis in promoting gastric tumorigenesis.

## Background

Gastric cancer (GC) is one of the most frequently diagnosed malignancies with poor prognosis worldwide, and the most common gastrointestinal malignancy in East Asia [1, 2]. According to data from GLOBOCAN 2018 (https://www.uicc.org/news/new-global-cancer-data-globocan-2018), gastric cancer is the 5th most common neoplasm and the 3rd most deadly cancer, with an estimated 783,000 deaths in 2018 [3]. In spite of the progress in radiotherapy, chemotherapy, and surgical techniques on GC patients, the survival rate of GC remains unsatisfactory [4, 5]. Recently, several oncogenes or tumor suppressors have been identified as key regulators in GC, however almost no commonly accepted biomarkers and therapy targets have been established to facilitate the management of GC patients [6]. Therefore, the identification of the novel regulators for gastric carcinogenesis will be of great importance to improve our understanding of GC.

Alternative splicing of pre-mRNA transcripts is an important process by which genomic complexity is generated from the relatively lower number of genes. By estimation, about 90% of human genes could produce alternatively spliced forms [7, 8]. The pre-mRNA splicing process is regulated by different splicing regulators, and their deregulations often result in aberrantly spliced individual variants and aberrant gene expression profiles. Intensive studies on splice variants have revealed that aberrant splicing contributes to a number of human diseases including tumorigenesis. For example, the splicing factor Serine and Arginine Rich Splicing Factor 2 (SRSF2) is upregulated frequently in human hepatocellular carcinoma (HCC), resulting in poor prognosis in patients [9]. NIMA Related Kinase 2 (NEK2) promotes aerobic glycolysis through regulating splicing of Pyruvate Kinase M1/2 (PKM) and increasing the PKM2/PKM1 ratio in myeloma cells which contributes to its oncogenic activity [10]. Splicing Factor 3b Subunit 3 (SF3B3) controlled the alternative splicing of Enhancer of Zeste 2 Polycomb Repressive Complex 2 Subunit (EZH2) pre-mRNA and contributed to the tumorigenic potential of renal cancer [11]. Moreover, Matos et al. [12] found that RAC1b, an alternative splice variant of the Rac Family Small GTPase 1 (RAC1), was increased in colorectal tumors and its high expression was required to sustain tumor cell viability. Additionally, the overexpression of CD44v6, an alternative splice variant of the CD44S, was accompanied by the upregulation of genes involved in epithelial-mesenchymal transition (EMT), metabolism and angiogenesis in gastric cancers [13, 14].

CD44 is a membrane receptor for hyaluronic acid, the major component of the extracellular matrix. CD44 gene is encoded by at least 20 exons, which generates several isoforms through extensive alternative splicing [13, 15, 16]. Only the 10 constitutively spliced exons are transcribed in the standard form (CD44S), while additional 10 variant exons (v1–v10) between construct exon 5 and 15 could be alternatively spliced in a very large number of different combinations [15]. CD44 and its spliced isoforms are known to be of central roles in the regulation of cellular behavior such as cell survival, growth and motility. Thus, the alternative splicing of CD44 is often deregulated in cancers, and produce various isoforms with properties that may have different tissue specific effects and therefore even diverse effects on cancer progression [16, 17]. In addition, the expression of CD44 isoforms has been reported to be under the control of several proteins such as Serine and Arginine Rich Splicing Factor 1 (SRSF1) and c-Fos [18, 19]. Therefore, it is of great importance to identify the functions of CD44 splicing isoforms, and to investigate the mechanisms of alternative splicing of CD44 in different cancers. Although the function of diverse CD44 isoforms has been characterized in multiple cancers recently, the mechanisms that responsible for the splicing of different CD44 isoforms is relatively less known.

Heterogeneous nuclear ribonucleoprotein K (hnRNPK) belongs to the DNA/RNA binding hnRNP family. hnRNPK has been found to shuttle between nucleus and cytoplasm, and its molecular function has been reported to be associated with gene transcription, pre-mRNA splicing, mRNA nuclear export, mRNA translation and decay [20–24]. In this study, we demonstrated that the expression of hnRNPK was significantly increased in GC and its upregulation was associated with tumor stage and metastasis. We also uncovered that hnRNPK activates the expression of the splicing regulator SRSF1. The serine/arginine (SR) protein family is an important class of splicing regulators and its members, including SRSF1, SRSF3, and SRSF6, have shown multiple proto-oncogenic properties and aberrant expressions in various cancer cells [25–27]. A recent study has indicated that SRSF1 could promote the splicing of CD44V6 (V6 exon-containing isoform) splicing in breast cancer cells [18]. Here we found that as a consequence, SRSF1 increased splicing activity and expression of an oncogenic CD44 isoform, CD44E (which has additional variant exons 8 to 10, CD44v8-v10) [28]. These findings revealed the importance of the hnRNPK-SRSF1-CD44E axis in promoting gastric tumorigenesis.

## Materials and methods

### Cell culture

The MGC-803 cells used in this study were obtained from the ATCC and cultured in DMEM medium supplemented with 10% fetal bovine serum and antibiotics (100 U/mL of penicillin and 100 mg/L of streptomycin). Cells were grown in a 5% CO_2_ atmosphere at 37 °C.

### Analysis of TCGA data

The GC microarray and RNA-seq data were downloaded from The Cancer Genome Atlas database (http://cancergenome.nih.gov). The extraction files were imported into Partek Genomic Suite Software (Partek Inc., Chesterfield, MO, USA). Gene expression data were normalized and log2 transformed. Then principal component analysis was performed to identify outliers and artifacts on the microarray. After quality check, the one-way analysis of variance (ANOVA) model using the method of moments was applied to identify differentially-expressed genes between tumor and control group or between neoplasm histologic stages (Grade 1–2 vs. Grade 3–4) of patients or between pathologic T stages (T1–2 vs. T3–4) with the Fisher’s least significant difference (LSD) contrast method.

### RNA isolation and qRT-PCR analysis

Total RNA was extracted from cells by using Trizol reagent (Invitrogen, CA, USA) according to the manufacturer’s instructions. qRT-PCR analysis was performed to detect the level of RNA transcripts. In brief, cDNA was synthesized by M-MLV reverse transcriptase (Invitrogen) from 4 μg of total RNA. Oligo (dT18) RT primer was used for the reverse transcription of mRNA. RT-qPCR was performed on the Bio-rad CFX96 real-time PCR system (Bio-rad, Foster City, CA, USA) using TB Green Fast qPCR Mix (TAKARA, Dalian, China) with the following cycling conditions: 95 °C for 1 min (initial denaturation), followed by 40 cycles of 95 °C for 15 s, 60 °C for 60 s. GAPDH was used for mRNA normalization. Primer sequences are listed in Additional file 1: Table S1.

### Oligonucleotides and constructs

The shRNA specific to hnRNPK and control shRNAs (shRNA-control) were synthesized by Dharmacon (GE Healthcare, Lafayette, CO, USA) and transfected (100 nM) using DharmFECT1. For hnRNPK or SRSF1 overexpression, the human SRSF1 cDNA ORF was inserted into the pcDNA3.1 vector (pCDNA-SRSF1). Transfection of the constructs was carried out with Lipofectamine 2000 (Invitrogen, Carlsbad, CA, USA) for GC cells according to the manufacturer’s protocols. The promoter-luciferase reporter constructs were generated by cloning PCR-amplified DNA fragments of human IFN1 promoter upstream of the promoter less firefly luciferase gene in the pGL3-basic vector (Promega, Madison, WI, USA). The MGC-803 cells were transfected using Lipofectamine-2000 reagent and luciferase activity in cell lysate was measured as previously described. The results were normalized for the transfection efficiency as relative to light units per Renilla luciferase activity.

### Cell proliferation assay

MGC-803 cells were incubated in 10% CCK-8 (DOJINDO, Japan) diluted in normal culture medium at 37 °C until visual color conversion occurred. Proliferation rates were determined at 0, 12, 24, 36, 48, 60, 72 and 96 h after transfection. The absorbance of each well was measured with a microplate reader set at 450 and 630 nm. All experiments were performed in triplicate.

### Cell migration and invasion assays

MGC-803 cells were grown to confluence on 12-well plastic dishes and treated with siRNAs or control. At 24 h after transfection, linear scratch wounds (in triplicate) were created on the confluent cell monolayers using a 200 μL pipette tip. To remove cells from the cell cycle prior to wounding, cells were maintained in serum-free medium. To visualize migrated cells and wound healing, images were obtained at 0, 24, and 48 h. Ten areas were selected randomly from each well, and the cells in three wells of each group were quantified.

For the invasion assays, after 24 h of transfection, 1 × 10^5^ MGC-803 cells in serum-free media were seeded onto the transwell migration chambers (8 μm pore size; Millipore, Switzerland), in which the upper chamber of an insert was coated with Matrigel (Sigma-Aldrich, USA). Media containing 20% FBS were added to the lower chamber. After 24 h, the non-invading cells were removed with cotton wool. Invasive cells located on the lower surface of the chamber were stained with May–Grunwald–Giemsa stain (Sigma-Aldrich, USA) and counted using a microscope (Olympus, Tokyo, Japan). Experiments were independently repeated three times.

### Immunoblotting analysis

Whole-cell lysate or nuclear extract was subjected to immunoblotting analysis using standard methods. Proteins were separated by 10% SDS-PAGE and transferred onto PVDF membranes (Millipore Corporation, Billerica, MA, USA). Membranes were blocked overnight with 5% non-fat dried milk for 2 h and incubated with anti-hnRNPK (1:1000) or SRSF1 (1:1000) antibody overnight at 4 °C. After washing with TBST (10 mM Tris, pH 8.0, 150 mM NaCl, and 0.1% Tween 20), the membranes were incubated for 2 h at room temperature with goat anti-rabbit antibody (Zsgb-bio, Beijing, China). SRSF1 antibody (#32-4500) was purchased from Thermo Fisher Scientific, and hnRNPK antibody (ab39975) was purchased from Abcam. All the experiments were repeated at least once with similar results. ImageJ software was used to quantify the Western blot results.

### Chromatin immunoprecipitation (ChIP)

ChIP was performed with MGC-803 cells in at least two independent experiments. Cells were chemically cross-linked by the addition of a one-tenth volume of fresh 11% formaldehyde solution for 15 min at room temperature, then homogenized, resuspended, lysed in lysis buffers, and sonicated to solubilize and shear crosslinked DNA into 150–250 base-pair (bp) segments. The resulting whole-cell extract was incubated overnight at 4 °C with 100 μl of Dynal Protein G magnetic beads that had been pre-incubated with 10 μg of the appropriate antibody. Beads were washed five times with RIPA buffer and once with TE (10 mM Tris–HCl pH 7.5, 1 mM EDTA) containing 50 mM NaCl. Bound complexes were eluted from the beads by being heated at 65 °C with occasional vortex-mixing, and crosslinking was reversed by incubation overnight at 65 °C. Whole-cell extract DNA (reserved from the sonication step) was also treated for crosslink reversal. Immunoprecipitated DNA and whole-cell extract DNA were then purified by treatment with RNaseA, proteinase K and multiple extractions with phenol/chloroform/3-methylbutan-1-ol. Purified DNA samples were normalized and subjected to PCR analysis. Antibodies used for pulldowns was anti-hnRNPK (ab39975) from Abcam. After immunoprecipitation, recovered chromatin fragments were subjected to semiquantitative PCR or real-time PCR for 32–40 cycles, using primer pairs specific for 150–250 bp segments (Additional file 1: Table S2.

### Statistics

Each experiment was repeated at least three times. Student’s t-test (two-tailed) was performed and three-group data were analyzed using one-way ANOVA. All statistical analyses were performed using SPSS 16.0 software (SPSS Inc., Chicago, IL, USA). Statistically significance was set at p < 0.05.

## Results

### The expression of hnRNPK is upregulated in GC patients

**Fig. 1:**
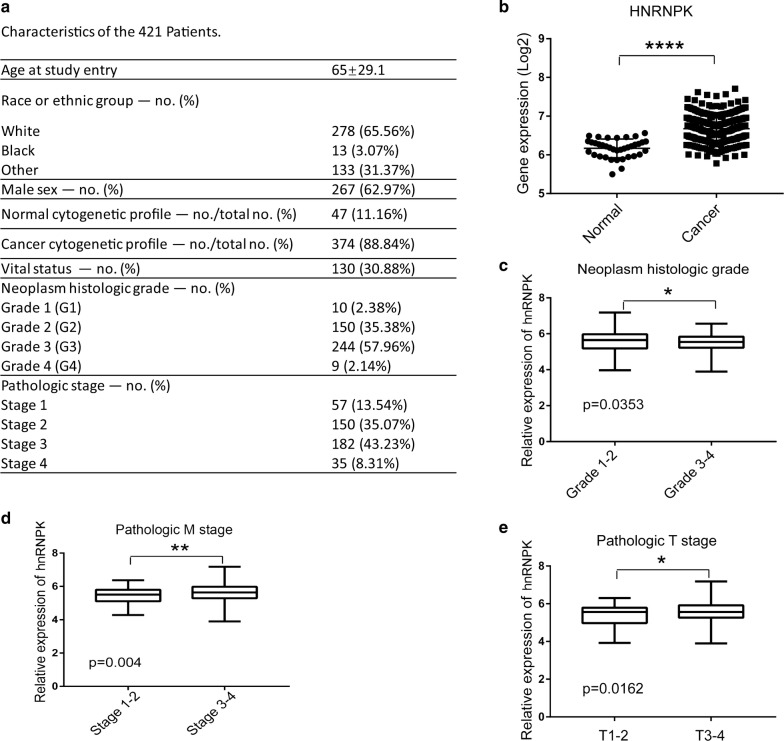
hnRNPK is upregulated in GC tissues. **a** GC patients information from TCGA database. **b** Relative hnRNPK mRNA level in GC and normal tissues from TCGA database. **c** Relative hnRNPK expression level in tissues from different neoplasm histologic grades of GC (Grade 1 and 2 vs. Grade 3 and 4). **d** Relative hnRNPK expression level in GC patients with different pM stage (metastasis vs. non-metastasis). **e** Relative hnRNPK expression level in GC patients with different pT stage (T1 and 2 vs. T3 and 4). Statistical analysis is described in “Materials and methods”. *p < 0.05; **p < 0.01; ****p < 0.0001

We first analyzed the expression pattern of hnRNPK mRNA in a total of 374 clinical samples of GC and 47 normal controls in TCGA (the Cancer Genome Atlas) datasets. We observed that hnRNPK was significantly upregulated in GC tissues compared with the normal counterparts (Fig. 1a, b), indicating the oncogenic role of hnRNPK in GC. However, hnRNPK expression was decreased in tissues from higher neoplasm histologic grades of GC (Fig. 1c, *p *=* 0.03*, Grade 3 and 4 vs. Grade 1and 2), suggesting that hnRNPK expression is positively correlated with well-differentiated GC cells. Notably, we found that a higher hnRNPK level was associated with GC pM stage (Fig. 1d, *p *= 0.004, metastasis vs. non-metastasis), and GC pT stage (Fig. 1e, *p *= 0.01, T3 and 4 vs. T1 and 2). These results indicated that the upregulation of hnRNPK in GC patients might function as a carcinogenic stimulus in GC tumorigenesis.

### Knockdown of hnRNPK inhibits GC cell proliferation, migration and invasion

**Fig. 2:**
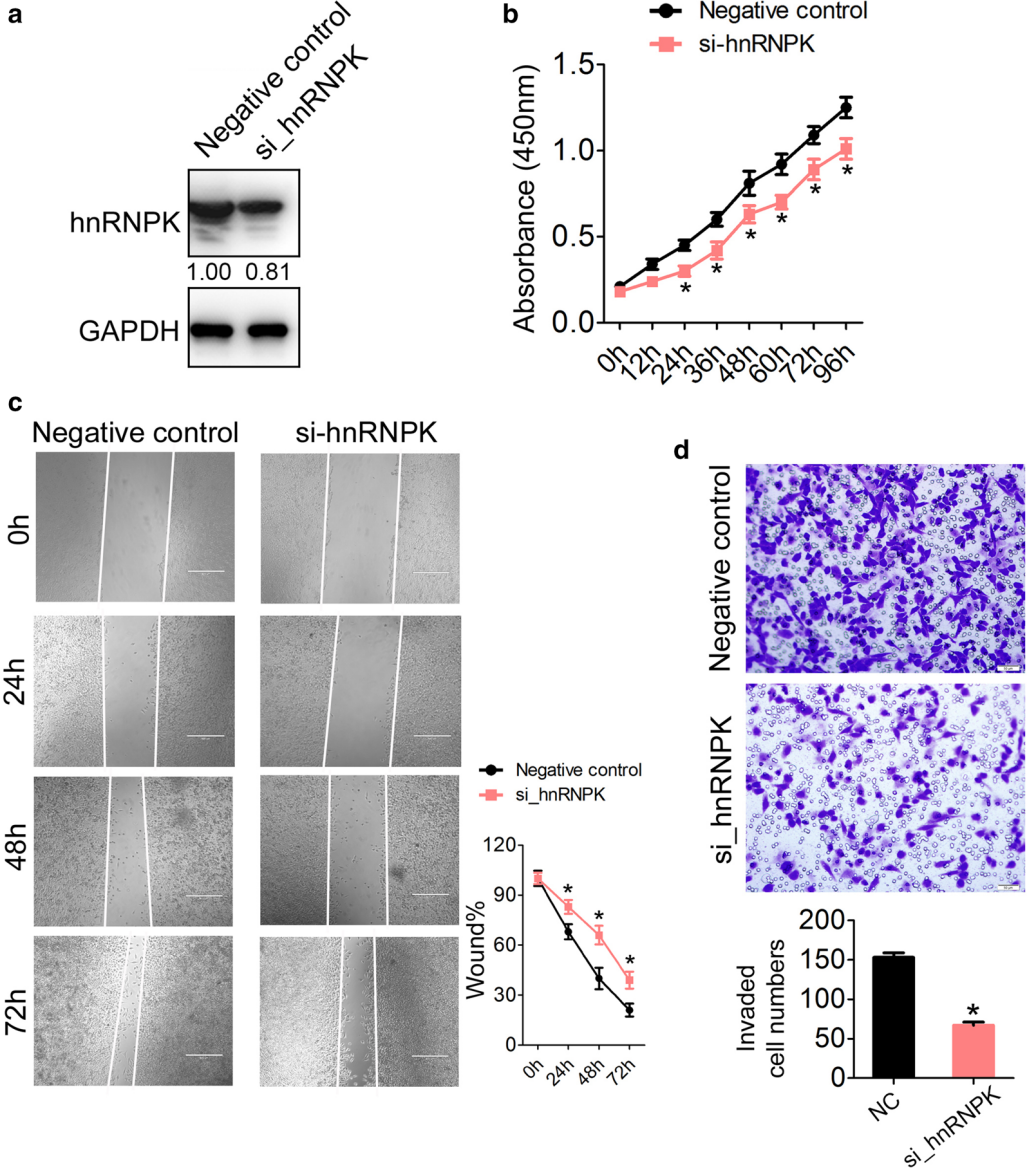
Knock-down of hnRNPK inhibits GC cell proliferation, migration and invasion. **a** The hnRNPK protein levels were detected in MGC-803 cells after treated by siRNA control or siRNA to hnRNPK (si_hnRNPK) by immunoblotting. **b** Cell proliferation assay of MGC-803 cells after transfected with si_control or si_hnRNPK using CCK-8. **c** Wound healing assays of MGC-803 cells after transfected with si_control or si_hnRNPK. The relative ratio of wound closure per field was shown in the right. **d** Transwell analysis of MGC-803 cells after transfected with si_control or si_hnRNPK. The relative ratio of invasive cells per field is shown below. The data are presented as the mean ± SEM, and the error bars represent the standard deviation obtained from three independent experiments. *p < 0.05

To investigate the functional significance of hnRNPK in the pathogenesis of GC, we used siRNAs specific to hnRNPK (si_hnRNPK) and scrambled oligonucleotides (Negative control) to transfect into GC cell line MGC-803. The efficiency of hnRNPK knockdown was confirmed by immunoblotting (Fig. 2a). The intracellular hnRNPK protein level was reduced by twofold in MGC-803 cells treated with si_hnRNPK than the negative control (Fig. 2a). CCK-8 results indicated that MGC-803 cells with decreased hnRNPK expression showed a significantly slower proliferation rate than control (Fig. 2b). Moreover, wound healing assay showed that cell migration was also reduced in hnRNPK-reduced MGC-803 cells compared with the control (Fig. 2c). Additionally, transwell invasion assay revealed a significant reduction in cell invasiveness after hnRNPK knockdown in MGC-803 cells (Fig. 2d). Taken together, these results suggested that hnRNPK might act as an oncogene in gastric carcinogenesis.

### hnRNPK binds to SRSF1 promoter and activates its transcription in GC cells

**Fig. 3:**
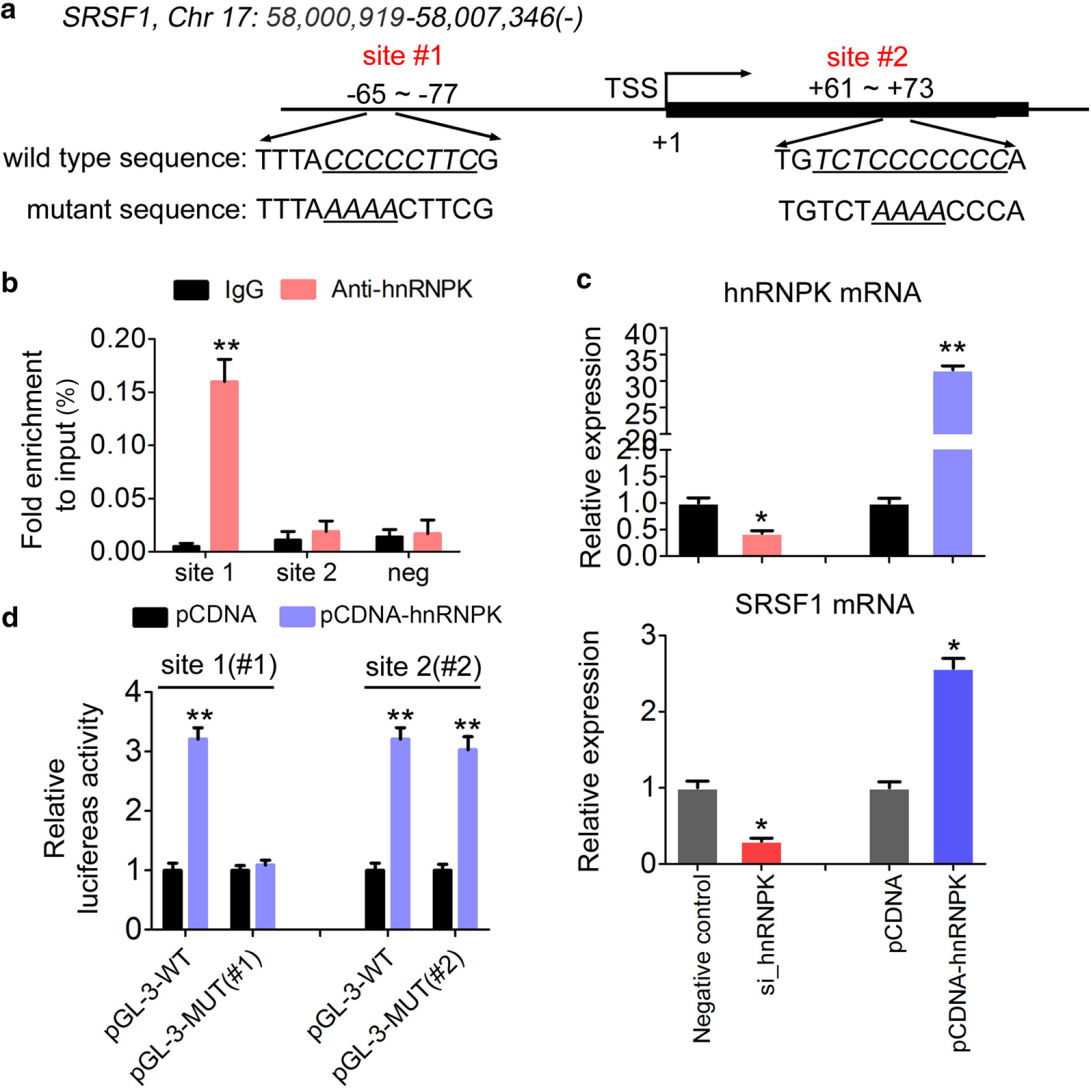
hnRNPK promotes the transcription of SRSF1 by binding to its promotor. **a** Schematic diagram showing the two putative hnRNPK binding motifs within the promoter region of human SRSF1 loci. **b** Quantitative ChIP-PCR (ChIP-qPCR) was performed using hnRNPK antibody in GC-803 cells. The negative IP was performed using anti-rabbit IgG. **c** Relative expression of hnRNPK (upper) and SRSF1 (lower) expression in MGC-803 cells after transfection with si_control or si_hnRNPK, pcDNA or pcDNA-HNRNPK. **d** The relative luciferase activities in MGC-803 cells after transfected with pcDNA or pcDNA-hnRNPK. The cells were co-transfected with either a wild type pGL-3-promoter construct (WT) or a mutant promoter (MUT) of SRSF1. For all quantitative results, the data are presented as the mean ± SEM, and the error bars represent the standard deviation obtained from three independent experiments. *p < 0.05; **p < 0.01

As a transcription factor, hnRNPK tends to bind a C-rich CT element sequence, as discovered in multiple gene promoters. By bioinformatics analysis, we found two putative hnRNPK binding motifs scattered within the promoter region of human SRSF1 loci (Fig. 3a). Quantitative ChIP-PCR (ChIP-qPCR) analysis was used to validate promoter binding of hnRNPK and the results showed that only the − 65 site (site 1) had hnRNPK occupancy in MGC-803 cells (Fig. 3b). To further determine whether hnRNPK could influence the expression of SRSF1, SRSF1 mRNA level was evaluated in MGC-803 cells transfected with siRNA specific to hnRNPK or constructs overexpressing hnRNPK (Fig. 3c). Accordingly, inhibition of HNRNPK repressed SRSF1 by threefold (Fig. 3c), whereas overexpression of hnRNPK enhanced the levels of SRSF1 (Fig. 3c). To confirm the activity of hnRNPK on SRSF1 promoter, we performed luciferase assay following co-transfection with an hnRNPK overexpressing-vector and either a wildtype pGL3-promoter construct (WT) or a mutant promoter (MUT) in MGC-803 cells (Fig. 3d). As expected, the increased hnRNPK levels successfully increased reporter activity by threefold. However, introduction of a mutation to site 1 not site 2 abolished this activity (Fig. 3d). These results suggested that hnRNPK directly binds to SRSF1 promoter and activates its transcription.

### The oncogenic role of hnRNPK is mediated by SRSF1 in GC cells

**Fig. 4:**
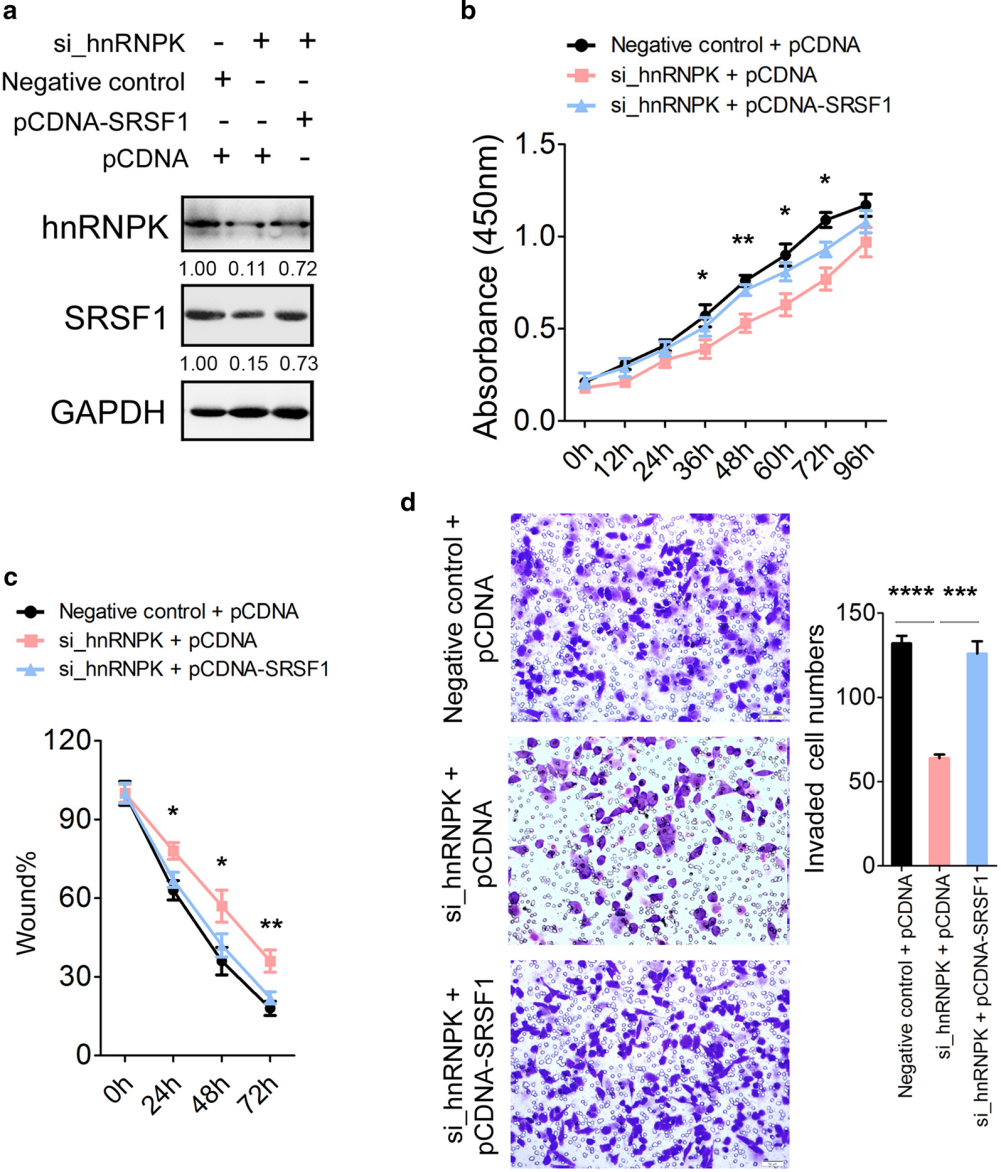
Rescue assays in MGC-803 cells. **a** Expression of hnRNPK and SRSF1 protein in MGC-803 cells after co-transfection with si_hnRNPK and pCDNA-SRSF1 by immunoblotting. **b** Cell proliferation assay of MGC-803 cells after transfected with si_hnRNPK and pCDNA-SRSF1 using CCK-8. **c** Wound healing assays of MGC-803 cells after transfected with si_hnRNPK and pCDNA-SRSF1. **d** Transwell analysis of MGC-803 cells after transfected with si_hnRNPK and pCDNA-SRSF1. The relative ratio of invasive cells per field is shown in the right. The data are presented as the mean ± SEM, and the error bars represent the standard deviation obtained from three independent experiments. * Represents si_hnRNPK + pCDNA compared with si_hnRNPK + pCDNA-SRSF1 in **b** and **c**. *p < 0.05; **p < 0.01; ***p < 0.001; ****p < 0.0001

To further determine whether the oncogenic role of hnRNPK is directly mediated by SRSF1, we performed rescue assay by co-transfection with siRNA specific to hnRNPK (si_hnRNPK) and a construct containing SRSF1 ORF (pCDNA-SRSF1) into MGC-803 cells. After rescue, a 2- to threefold increase in SRSF1 protein levels was observed in MGC-803 treated with the combination of si_hnRNPK and pCDNA-SRSF1 compared to the transfection combination of si_hnRNPK and pCDNA (Fig. 4a). Consequently, this led to an increase in cell proliferation (Fig. 4b), migration (Fig. 4c) and invasion (Fig. 4d) in MGC-803 cells. Thus, the reintroduction of SRSF1 into GC cells could rescue the cellular phenotype caused by hnRNPK knockdown, which indicated that the oncogenic role of hnRNPK is mediated by SRSF1 in gastric carcinogenesis.

### hnRNPK regulates the alternative splicing of CD44 through SRSF1

**Fig. 5:**
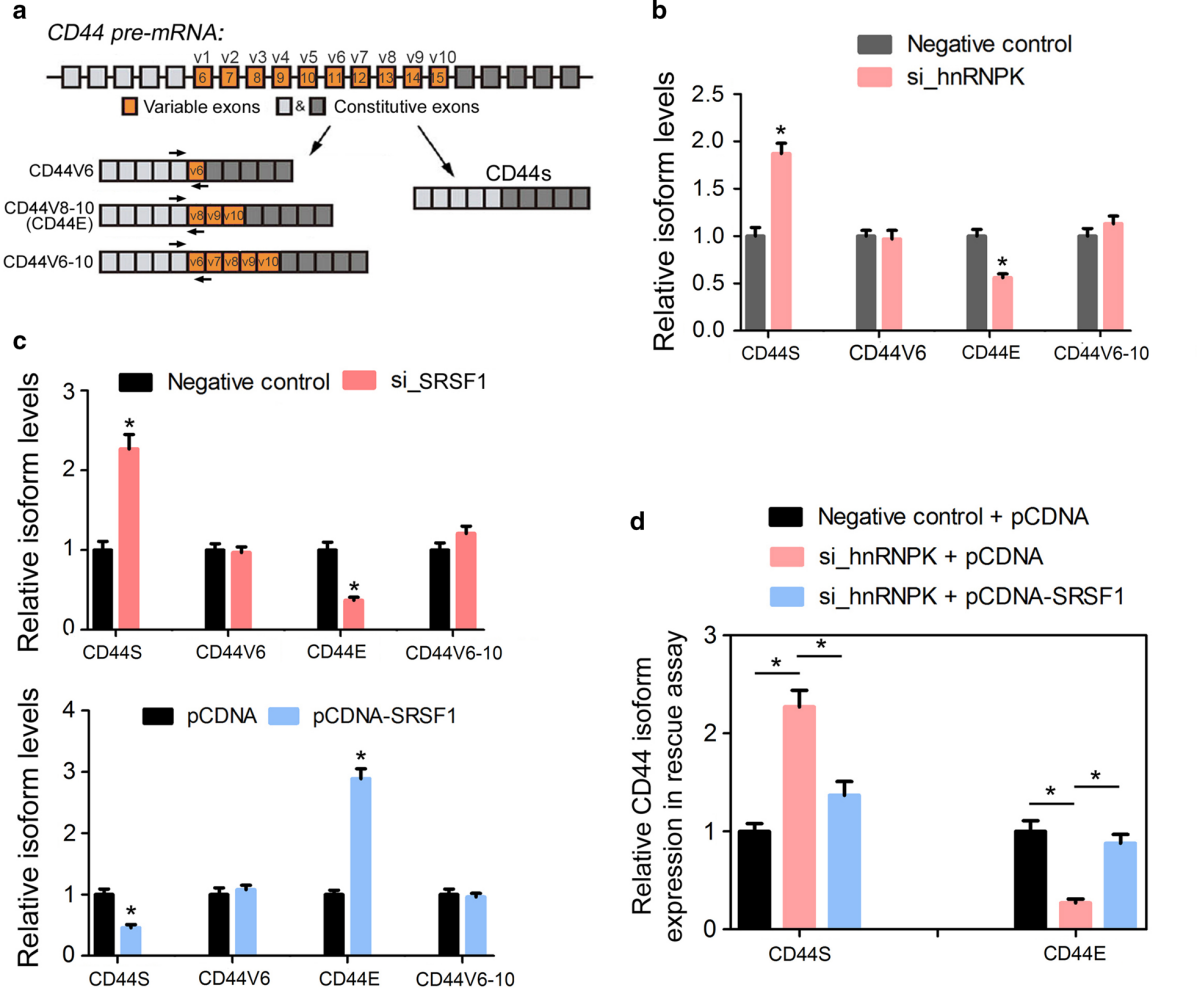
hnRNPK regulates the alternative splicing of CD44 through SRSF1. **a** Schematic diagram showing the different splicing variants of CD44 (full-length CD44), CD44V6 (only contains variable exon 6), CD44V8-10 (contains variable exons 8–10) CD44V6-10 (contains variable exons 6–10) and CD44S (has no variable exons) isoforms. The arrows indicate the location of specific primer sets designed for qRT-PCR analysis of different IRF3 splicing variants. **b** Relative expression of CD44 splicing variants in MGC-803 cells after transfected with si_control or si_HNRNPK. **c** Relative expression of CD44 splicing variants in MGC-803 cells after transfected with si_control or si_SRSF1 (upper), or pCDNA or pCDNA-SRSF1 (lower). **d** Relative expression of CD44 splicing variants in MGC-803 cells after transfected with after co-transfection with si_hnRNPK and pCDNA-SRSF1 or their negative controls. For all quantitative results, the data are presented as mean ± SEM, and the error bars represent the standard deviation obtained from three independent experiments. *p < 0.05

To test whether SRSF1 could regulate the alternative splicing of CD44 in GC cells, as it was reported in breast cancer cells [18], we detected the relative levels of different CD44 isoforms in MGC-803 cells transfected with siRNA specific to SRSF1 or negative control (Fig. 5a). The changes in SRSF1 protein levels led to a significant decrease in CD44E isoform levels, but an increase in CD44S levels. No obvious changes were observed in CD44V6 and CD44V6-10 isoforms (Fig. 5b). Conversely, over expression of SRSF1 in MGC-803 cells specifically upregulated CD44E levels in the expense of CD44S expression (Fig. 5b). This result was consistent with a previous report that CD44E was the major variant transcript of CD44 in gastric cancer cells [15]. To further investigate whether hnRNPK could also affect the expression of CD44 isoforms via its regulation on SRSF1, we next detect the changes of CD44 isoforms upon hnRNPK knockdown in MGC-803 cells. As expected, when hnRNPK was reduced in MGC-803, CD44E level was accordingly repressed but CD44S was upregulated (Fig. 5c). Furthermore, the reintroduction of SRSF1 upon hnRNPK knockdown in MGC-803 cells could rescue the expression of CD44E, suggesting the existence of hnRNPK-SRSF1-CD44E axis in GC.

## Discussion

The classification of a specific gene as oncogene or tumor suppressor has been a staple of cancer research. However, this simple classification has become increasingly difficult for some genes [29]. hnRNPK is one of the confused genes. Its tumor suppressor role has recently been described in acute myeloid leukemia and demonstrated by a haploinsufficient mouse model [22]. In contrast, data from other clinical correlation studies suggest that hnRNPK may be more fittingly described as an oncogene, due to its increased levels in a variety of malignancies [23, 29–33]. hnRNPK itself is a multifunctional protein that might regulate both oncogenic or tumor suppressive pathways through its diverse activates. In this study, we revealed that hnRNPK has an oncogenic role in gastric carcinogenesis by promoting cell proliferation, cell migration and invasion. Our findings highlight the current understanding of hnRNPK in tumorigenesis.

Our study also revealed that the splicing pattern of CD44 is controlled by hnRNPK in a SRSF1-dependent manner in GC. The ubiquitously expressed CD44 is a cell surface glycoprotein, which participates in cell–cell or cell-extracellular matrix interactions [15]. CD44 has been known to be related to tumorigenicity and regulates cell migration, invasion and metastasis [34–36]. It is well known that the alternative splicing of CD44 pre-mRNA is a main source of the diverse CD44 isoforms, and these isoforms with different properties might have diverse effects on cancer progression. The overexpression of CD44v9 has been associated with invasive prostate cancer and gastric cancer [37–39]. Another study indicated that the expression of CD44v6, in sporadic gastric tumors is a potential marker to distinguish intestinal- and diffuse-type gastric adenocarcinomas [40]. Moreover, CD44v8-v10 (CD44E) deregulation has been reported to be a prognostic marker in gallbladder cancer [41]. Serine and arginine-rich (SR) proteins are a protein family that includes 13 members, which have a common RNA recognition motif (RRM) domain and a RS domain. SR proteins have essential roles in alternative and constitutive splicing via promoting exon inclusion or skipping through interactions with specific RNA motifs in exons or introns. A previous study has performed a SR protein screen for CD44v6 splicing using overexpression and lentivirus-mediated shRNA treatment, which demonstrated that SRSF3 and SRSF4 have no obvious effects on V6 splicing, whereas SRSF1, SRSF6 and SRSF9 could significantly promote V6 splicing and favor the biogenesis of CD44V6 [28]. Here, we demonstrated that SRSF1 increases the splicing activity and expression of CD44E in GC cells. This novel finding might yield insights into the understanding of CD44 alternative splicing mechanism in tumorigenesis.

## Conclusion

hnRNPK was significantly increased in GC and associated with tumor stage and metastasis. Mechanistically, it increases the splicing activity and expression of a CD44 isoform, CD44E, to promote gastric tumorigenesis. Taken together, our results underscored the importance of the hnRNPK-SRSF1-CD44E axis in regulating gastric carcinogenesis.

## Supplementary information

**Additional file 1: Table S1.** Primers used for qRT-PCR. **Table S2.** Primers used for ChIP-qPCR.

## Data Availability

Not applicable.
