# Supplementary material for: hnRNPK promotes gastric tumorigenesis through regulating CD44E alternative splicing
Source: Cancer Cell Int. 2019 Dec 12;19:335. doi: 10.1186/s12935-019-1020-x (PMC6909542; doi:10.1186/s12935-019-1020-x)
Supplement: Supplementary file 1 — Additional file 1: Table S1. Primers used for qRT-PCR. Table S2. Primers used for ChIP-qPCR. [file 12935_2019_1020_MOESM1_ESM.docx]

Table S1. Primers used for qRT-PCR.

| Name | mRNA sequence accession number | Sequence (5’-3’) |
| --- | --- | --- |
| HNRNPK forward | NM_031262.3 | TAAAATCAAAGAACTTCGAGAGAAC |
| HNRNPK reverse |  | AGAACAACTCTGTCAGTGGAATGA |
| SRSF1 forward | NM_006924.5 | ATGTCGGGAGGTGGTGTGATTC |
| SRSF1 reverse |  | TGTTCCACGGCCGCTTCGAG |
| CD44S forward | NM_001202556.2 | AATCCCTGCTACCAGAGACC |
| CD44S reverse |  | TTCAGATCCATGAGTGGTATGGG |
| CD44V6 forward | NM_001202557.2 | AGGAACAGTGGTTTGGCAAC |
| CD44V6 reverse |  | CGAATGGGAGTCTTCTCTGG |
| CD44E forward | NM_001202555.2 | GACAAGTTTTGGTGGCACG |
| CD44E reverse |  | CACGTGGAATACACCTGCAA |
| CD44V6-10 forward | NM_001001390.2 | AGGAACAGTGGTTTGGCAAC |
| CD44V6-10 reverse |  | CTGGAGTCCATATCCATCCTTC |
| GAPDH forward | NM_001289746.2 | TCAACGACCACTTTGTCAAGCTCAGCT |
| GAPDH reverse |  | GGTGGTCCAGGGGTCTTACT |

Table S2. Primers used for ChIP-qPCR.

| Name | Sequence (5’-3’) | Length (bp) |
| --- | --- | --- |
| Site_1 forward | CGACCTGGATGACTAGCTCCT | 165 |
| Site_1 reverse | CTACGTTCAGCGCACGC |  |
| Site_2 forward | GTGCGTGTTGGGATCGAAT | 212 |
| Site_2 reverse | CACACCACCTCCCGACAT |  |
| Neg forward | CACTGCGACTAGCACTAGGAGT | 135 |
| Neg reverse | TGGCTGCTTAGACAGATGTGTCT |  |
